# Veränderungen sexueller Interessen und Erfahrungen während der COVID-19-Pandemie - Eine qualitative Inhaltsanalyse

**DOI:** 10.1007/s00278-021-00506-5

**Published:** 2021-03-30

**Authors:** Johanna Schröder, Emily Bruns, Wiebke Schoon, Peer Briken, Daniel Schöttle

**Affiliations:** 1 Institut für Sexualforschung, Sexualmedizin & Forensische Psychiatrie, Universitätsklinikum Hamburg-Eppendorf, Martinistr. 52, 20246 Hamburg, Deutschland; 2 Klinik und Poliklinik für Psychiatrie und Psychotherapie, Universitätsklinikum Hamburg-Eppendorf, Martinistr. 52, 20246 Hamburg, Deutschland

**Keywords:** Sexuelle Gesundheit, Physische Distanzierung, Soziale Beziehungen, Dating, Intimität, Sexual health, Physical distancing, Social relationships, Dating, Intimacy

## Abstract

**Hintergrund:**

Die Kontaktbeschränkungen zur Eindämmung der durch die „coronavirus disease 2019“ (COVID-19) ausgelösten Pandemie beeinflussen das soziale Leben der Menschen auf vielen Ebenen, so auch die psychische und sexuelle Gesundheit.

**Fragestellung:**

Ziel der Studie war es, Veränderungen sexueller Interessen und Erfahrungen in Deutschland lebender Personen während der ersten Kontaktbeschränkungen im Frühjahr 2020 zu erfassen.

**Material und Methoden:**

In einer anonymen Online-Befragung wurde eine offene Frage nach Veränderungen der Sexualität durch die Kontaktbeschränkungen gestellt und mithilfe der qualitativen Inhaltsanalyse nach Mayring ausgewertet.

**Ergebnisse:**

Veränderungen wurden von 248 der teilnehmenden Personen in den Bereichen „sexuelles Verlangen und Erregbarkeit“, „Partnerschaft“, „sexuelle Aktivitäten“, „sexuelle Interessen und Einstellungen“, „virtuelle sexuelle Aktivitäten“, „Dating“ und „sexuelle Probleme und Funktionsstörungen“ beschrieben.

**Schlussfolgerung:**

Die Kontaktbeschränkungen im Rahmen der COVID-19-Pandemie führten zu sexuellen und partnerschaftlichen Veränderungen, die in Sexual- und Paartherapiesettings beachtet werden sollten.

## Abstract (en)

**Background:**

Contact restrictions imposed to slow the spread of the coronavirus disease 2019 (COVID-19) pandemic affect people’s social lives at various levels as well as their mental and sexual health.

**Objective:**

The present study aimed to assess changes in sexual interests and experiences of residents in Germany during the first wave of social contact restrictions in early 2020.

**Material and methods:**

In an anonymous online survey, answers to an open question regarding changes in sexuality due to contact restrictions were collected and subjected to Mayring’s qualitative content analysis.

**Results:**

The changes reported by 248 participants could be subsumed under the categories “sexual desire and arousability”, “relationship”, “sexual activities”, “sexual interests and attitudes”, “virtual sexual activities”, “dating” and “sexual problems and dysfunctions”.

**Conclusion:**

Social distancing rules and restrictions brought about by the COVID-19 pandemic have led to changes in sexuality and relationships, which should be considered in sexual and couple therapy settings.

Zur Eindämmung des „severe acute respiratory syndrome coronavirus type 2“ (SARS-CoV-2) wurden weltweit Regeln zur Kontaktbeschränkung erlassen, die soziale Kontakte reduzierten und Aktivitäten außerhalb der Wohnung einschränkten. Zugleich bedeuteten die Einschränkungen für viele Personen einen Anstieg der zu Hause und online verbrachten Zeit, was bedeutende Auswirkungen auf zwischenmenschliche Kontakte haben kann. Veränderungen sexueller Interessen und Erfahrungen während der Pandemie sind Gegenstand dieser qualitativen Untersuchung.

## Einleitung

Seit März 2020 wurden in Deutschland weitreichende Maßnahmen zur Eindämmung der Pandemie durch SARS-CoV‑2 eingeführt und im weiteren Verlauf dem dynamischen Geschehen angepasst. Die Maßnahmen umfassten u. a. das Tragen von Mund-Nase-Masken, Abstands- und Kontaktregeln sowie die Schließung von Gastronomiebetrieben, in denen Menschen üblicherweise Kontakte pflegen. Hinzu kamen die sich hieraus ableitenden weitreichenden beruflichen (z. B. Umstellung auf Homeoffice), familiären (z. B. ungleiche Arbeitsbelastung für Eltern durch Kita- und Schulschließungen) und finanziellen Veränderungen (z. B. Kurzarbeit, Arbeitsplatzverlust). Es liegt nahe, dass derartige das Privatleben beeinflussende Maßnahmen und deren direkte und indirekte Folgen sich auf zwischenmenschliche Kontakte sowohl positiv wie auch negativ auswirken können. Es finden sich international erste Studien zu diesem Thema, die aufgrund methodischer Unterschiede zwar nur eingeschränkt miteinander vergleichbar sind, jedoch einen interessanten Einblick in die Auswirkungen von sozialen Einschränkungen geben, wie sie im Nachkriegsdeutschland bisher noch nie vorgekommen sind: In einer Online-Studie (*n* = 1588) mit hauptsächlich weiblichen, heterosexuellen und weißen US-Amerikaner_innen wurde im Erhebungszeitraum vom 21.03.2020 bis zum 14.04.2020 – gegen Ende März 2020 hatten mehr als die Hälfte der US-Staaten eine häusliche Isolation („stay-at-home order“) verhängt – von 44 % der Teilnehmer_innen eine Abnahme sexueller Aktivitäten seit Beginn der Pandemie berichtet (Lehmiller et al. 2020). Dieser Trend spiegelte sich in den Ergebnissen einer italienischen Studie (*n* = 89) an Probandinnen ca. 4 Wochen nach Einführung der „Social-distancing“-Regeln wider (Schiavi et al. 2020). In einer Stichprobe verheirateter türkischer Frauen (*n* = 58) wurden hingegen eine signifikante Zunahme der sexuellen Kontakte während der Pandemie (Erhebungszeitraum: 11.03.2020–12.04.2020) und gleichzeitig ein negativer bewertetes sexuelles Erleben im Vergleich zu 6 bis 12 Monaten vor der Pandemie festgestellt (Yuksel und Ozgor 2020). Vorläufige Ergebnisse einer Untersuchung englisch- und spanischsprachiger Stichproben (*n* = 279) im März und im April 2020 lieferten wiederum keine Hinweise auf eine durchschnittliche Veränderung der Frequenz von sexuellen Kontakten, wobei 10 % dieser Proband_innen eine erhöhte Masturbationsfrequenz berichteten (Ibarra et al. 2020). In einer internationalen Studie (*n* = 4813) aus 7 EU-Ländern und der Türkei von Mai bis Juli 2020 berichteten 53 % der teilnehmenden zusammenlebenden Personen in Partnerschaften keine Veränderungen ihres sexuellen Verlangens, 28,5 % von einer Steigerung und 18,5 % von einer Reduktion ihres sexuellen Verlangens, wobei sich dieses Muster ähnlich über die Ergebnisse der 8 teilnehmenden Länder zeigte (Stuhlhofer et al. in Revision). In der beschriebenen US-amerikanischen Studie wurde von neu adaptierten sexuellen Aktivitäten (z. B. Sexting, neue Stellungen) während der Kontaktbeschränkungen berichtet, was im Zusammenhang mit jüngerem Alter, eigenem Wohnraum, Stress und Einsamkeit sowie einer erlebten Verbesserung der eigenen Sexualität stand (Lehmiller et al. 2020). Veränderungen in den partnerschaftlichen und familiären Beziehungen wurden in einer spanischen, vornehmlich weiblichen Stichprobe (*n* = 407) während der ersten 3 Wochen des staatlich verordneten „Lockdowns“ (häusliche Quarantäne, geschlossene Bildungseinrichtungen und Ausgangssperren mit Ausnahme von Versorgung und Arbeit) überwiegend (62 %) als positiv erlebt, z. B. durch gesteigerte emotionale Intimität (Günther-Bel et al. 2020). Kinderlose Paare berichteten über eine bessere Partnerschaftsqualität als Paare mit Kindern, die eine bessere Beziehungsqualität zu ihren Kindern als zueinander beschrieben (Günther-Bel et al. 2020).

Häufige auf die „coronavirus disease 2019“ (COVID-19) bezogene Partnerschaftskonflikte (z. B. vermehrte Konflikte, Anspannung und Beziehungsprobleme) waren in einer US-amerikanischen Studie (*n* = 742) im April 2020 mit Abnahmen sexueller Aktivitäten sowie des Austausches von Zärtlichkeiten in der Partnerschaft assoziiert (Luetke et al. 2020).

Es wird vermutet, dass die Kontaktbeschränkungen im sozialen Leben zu einer Verlagerung sexueller Aktivitäten auf virtuelle Ebenen führen können und beispielsweise der Konsum pornografischen Materials ansteigt (weltweit um 11,6 % am 17.03.2020 im Vergleich zum Vorjahr; Pornhub 2020).

Die aktuelle Forschung zu den Themen Kontaktbeschränkungen und Sexualität präsentiert bislang zwar vielfältige, aber auch heterogene Ergebnisse. Ziel der aktuellen Studie ist es, die bisherigen quantitativen Studien um eine qualitative Untersuchung zu den Veränderungen sexueller Interessen und Erfahrungen während der COVID-19-bedingten Kontaktbeschränkungen in einer deutschen Stichprobe zu ergänzen.

## Methodik

### Studiendesign

Anhand einer anonymen Online-Befragung wurden die Studienteilnehmer_innen im Zeitraum von Mitte Mai bis Ende Juli 2020 zu Veränderungen ihrer sexuellen Interessen und Erfahrungen durch die Kontaktbeschränkungen im ersten „Lockdown“ von Mitte März bis Mitte April 2020 befragt. Die zugrunde liegende Studie wurde von der lokalen psychologischen Ethikkommission des Universitätsklinikums Hamburg-Eppendorf geprüft (Referenznummer: LPEK-0160). Die Ergebnisse des vorliegenden Beitrags basieren auf folgender Instruktion: „Abschließend möchten wir Sie bitten, einen (oder zwei) Sätze zu schreiben, die Veränderungen Ihrer sexuellen Interessen und Erfahrungen durch die Pandemie kurz zusammenfassen“.

In einem iterativen induktiven Verfahren wurden das Textmaterial in Microsoft Excel nach der Methode der inhaltlich-strukturierenden und quantifizierenden Qualitativen Inhaltsanalyse nach Mayring (2003) kategorisiert. Anhand der sequenziellen Durcharbeitung des Materials wurde zunächst eine Ebene an Unterkategorien und im zweiten Schritt eine Ebene an Oberkategorien erstellt. Die anschließende Quantifizierung der Kategorien erfolgte primär basierend auf der Häufigkeit der Nennung von Inhalten und sekundär auf der Fallebene, sodass das Zitat einer Person sich zwar in mehreren Kategorien wiederfinden konnte, es jedoch kein mehrfaches Auftauchen derselben Kategorie innerhalb einer Person gab. Als Bezugsgröße der Prozentzahlen dient die finale Stichprobe an Personen, die eine Veränderung berichten.

Zur Schätzung der Interrater-Reliabilität wurden mithilfe von RStudio, Version 1.3.959, anhand einer computergenerierten Zufallsauswahl 20 Zitate ausgewählt und 2 unabhängigen Rater_innen vorgelegt. Hierbei ergab sich ein Krippendorff’s α von .75.

### Stichprobe und Rekrutierung

Die Studienteilnehmer_innen wurden über soziale Medien, persönliche Kontakte, E‑Mail-Verteiler und Pressestellen rekrutiert. Einschlusskriterien waren die Vollendung des 18. Lebensjahrs und eine digitale informierte Einverständniserklärung.

## Ergebnisse

### Stichprobencharakteristika

Es machten 328 Personen gültige inhaltliche Angaben, wovon 80 (24,4 %) Proband_innen mitteilten, keine Veränderungen erlebt zu haben. Nach Ausschluss dieser Fälle umfasste die finale Stichprobe derer, die subjektiv Veränderungen in ihrem sexuellen Erleben und Verhalten wahrgenommen haben, 248 Personen (Tab. 1). Das durchschnittliche Alter dieser Stichprobe betrug 31 Jahre (Range: 18 bis 66 Jahre), und zwei Drittel der Teilnehmer_innen waren weiblich. Der Großteil der Teilnehmer_innen zeigte ein hohes Bildungsniveau, und fast die Hälfte war vollzeitig erwerbstätig. Drei Viertel der Teilnehmenden lebten in Großstädten. Die meisten Teilnehmer_innen teilten ihren Wohnraum mit anderen Personen. Etwa zwei Drittel der Stichprobe befanden sich zum Erhebungszeitpunkt in monogamen Partnerschaften, etwa ein Drittel war in keiner Partnerschaft, und ein kleinerer Teil der Stichprobe führte nichtmonogame Partnerschaften. Weniger als ein Viertel der Teilnehmer_innen hatten leibliche Kinder. Der Großteil der Proband_innen bezeichnete sich als ausschließlich oder überwiegend heterosexuell.

**Table 1:** 

	*N*	%
**Alter**		
*M* = 31,1 (*SD* = 9,4;* Mdn* = 30)		
**Geschlecht**		
Weiblich	164	66,1
Männlich	82	33,1
Divers	2	0,8
**Bildungsstatus**		
Noch kein Schulabschluss (Schüler_in)	2	0,8
Hauptschulabschluss	0	0,0
Realschulabschluss/mittlere Reife	28	11,3
Abitur/Fachhochschulreife	218	87,9
**Einwohnerzahl des Wohnorts**		
Landgemeinde	17	7,0
Klein- bis Mittelstadt	47	19,3
Großstadt	179	73,7
**Wohnsituation**		
Alleine lebend	65	26,2
Mit anderen zusammenlebend	176	71,0
Sonstiges/Mischformen	7	2,8
**Rückzugsmöglichkeiten in geteiltem Wohnraum (*n*** **=** **183)**		
Ja	150	82,0
Nein	33	18,0
**Beziehungsstatus**		
In keiner Partnerschaft/Single	75	30,2
In Partnerschaft (monogam bzw. exklusiv)	145	58,5
In Partnerschaft (nicht-monogam)	28	11,3
**Kinder**		
Ja (*M* = 1,8, *SD* = 0,6, *Mdn* = 2)	43	17,3
Nein	205	82,7
**Sexuelle Orientierung**		
Ausschließlich/überwiegend heterosexuell	205	82,7
Gleichermaßen heterosexuell wie homosexuell (bisexuell)	22	8,9
Überwiegend/ausschließlich homosexuell	14	5,6
Kein Interesse an sexuellen Reaktionen und Kontakten	0	0,0
Anders	7	2,8

*M* Mittelwert, *SD* Standardabweichung, *Mdn* Median

### Beschreibung des Textmaterials

Die Antworten auf die vorliegende Fragestellung umfassten im Durchschnitt 204 Zeichen (*Mdn* = 157 Zeichen, *SD* ± 157 Zeichen). Die kürzeste Texteingabe bestand aus 7 und die längste aus 906 Zeichen (einschließlich Leerzeichen). Die Teilnehmer_innen wählten größtenteils eine umgangssprachliche Ausdrucksweise und benutzten oftmals Abkürzungen, Teilsätze und Stichpunkte.

### Oberkategorien: Veränderungen sexueller Interessen und Erfahrungen

Die qualitative Inhaltsanalyse nach Mayring brachte 7 verschiedene Oberkategorien an Veränderungen sexueller Interessen und Erfahrungen hervor: „sexuelles Verlangen und Erregbarkeit“, „Partnerschaft“, „sexuelle Aktivitäten“, „sexuelle Interessen und Einstellungen“, „virtuelle sexuelle Aktivitäten“, „Dating-Verhalten“ und „sexuelle Probleme und Funktionsstörungen“. Im Folgenden werden diese näher beschrieben.

#### Sexuelles Verlangen und Erregbarkeit

Die am häufigsten benannten Veränderungen sexueller Interessen und Erfahrungen während der Covid-19-Pandemie bezogen sich auf „sexuelles Verlangen und/oder Erregbarkeit“ (Tab. 2). Von den Personen mit subjektiven Lustveränderungen berichtete etwas mehr als die Hälfte der Befragten eine Steigerung und etwas weniger als die Hälfte eine Reduktion des sexuellen Verlangens. Weiterhin zeichnete sich ein Anstieg des Verlangens nach Nähe, Intimität und/oder Körperkontakt ab, seltener auch eine diesbezügliche Aversion: „Anfangs [habe ich] körperliche Nähe zu anderen als unangenehm empfunden“ (Z5). Ein kleinerer Teil der Proband_innen gab eine Veränderung, zu drei Vierteln eine Zunahme, in der Masturbationsfrequenz an. Seltenere Angaben in dieser Oberkategorie bezogen sich auf gesteigerte Erregbarkeit („Zufällige leichte Berührungen … haben mich teilweise schon erregt, selbst wenn die Person gar nicht attraktiv für mich war“; Z111), eine Veränderung der Reize, die als erregend wahrgenommen werden („… vllt. einige spezifische Sachen wurden weniger attraktiv, die anderen eher mehr“; Z120) sowie die veränderte Bedeutung von Masturbation (zur Ablenkung und gegen Langeweile).

**Table 2:** 

	∑
**Veränderung des sexuellen Verlangens**	92
– 55,4 % Zunahme	
– 44,6 % Abnahme	
**Veränderung im Verlangen nach Nähe/Intimität/Körperkontakt**	29
– 90 % Zunahme	
– 10 % Abnahme	
**Veränderung in der Masturbationsfrequenz**	20
– 75 % Zunahme	
– 25 % Abnahme	
**Gesteigerte Erregbarkeit**	2
**Veränderung der Reize, die als anziehend wahrgenommen werden**	2
**Veränderung der Bedeutung von Masturbation**	1

#### Partnerschaft

Die zweithäufigste Oberkategorie thematisierte Veränderungen in Partnerschaften (Tab. 3) und vor allem die überwiegende Zunahme der gemeinsam verbrachten Zeit. Die Proband_innen berichteten ferner von Veränderungen in der emotionalen Intimität in ihrer Partnerschaft, insbesondere einer Steigerung dieser: „Durch die Kontaktbeschränkung, … der intensiveren Zeit zusammen, hat sich die emotionale Distanz um ein Vielfaches reduziert. Wir sind uns näher, was sich natürlich positiv auf die Beziehung und die Zuneigung zwischen einander auswirkt“ (Z156). Einige Befragte gaben an, eine neue monogame Beziehung eingegangen zu sein. Andere berichteten über ein gesteigertes Verlangen nach partnerschaftlichen Beziehungen oder von einem gesteigerten Interesse an Personen außerhalb der Partnerschaft bzw. fehlender Bestätigung von außen. Die Kontaktbeschränkungen führten einigen Berichten zufolge zu „zwangsweise monogamen“ Beziehungen und für eine Person zu reduziertem Kontakt mit ihren anderen Partner_innen in nichtmonogamen Beziehungsformen. Laut Bericht einer weiteren Person entwickelte sich eine Partnerschaft zu einer offenen Beziehung. Ein Teilnehmer berichtete Schuldgefühle, wenn er trotz der erhöhten Anwesenheit und dadurch sexuellen Verfügbarkeit seiner Partnerin masturbiert habe. Ein Fall von sexuellen Übergriffen in der Partnerschaft wurde berichtet.

**Table 3:** 

	∑
**Veränderung der mit dem/der Partner_in verbrachten Zeit**	38
– 76,3 % Zunahme	
– 23,7 % Abnahme	
**Veränderung in der emotionalen Intimität in der Partnerschaft**	20
– 75 % Zunahme	
– 25 % Abnahme	
**Neue monogame Beziehung**	8
**Gesteigertes Verlangen nach partnerschaftlichen Beziehungen**	8
**Gesteigertes Interesse an Personen bzw. fehlende Bestätigung außerhalb der Beziehung**	7
**Zwangsweise monogame Beziehung**	3
**Hürden, neue Partnerschaften einzugehen**	3
**Nicht mehr monogame Beziehung (z.** **B. offene Beziehung)**	1
**Weniger Kontakt zu anderen Partner_innen in nichtmonogamen Beziehungskonzepten**	1
**Schuldgefühle Partnerin gegenüber bei Masturbation**	1
**Sexuelle Übergriffe in der Partnerschaft**	1

#### Sexuelle Aktivitäten

Eine quantitative Veränderung (überwiegend eine Steigerung) zwischenmenschlicher sexueller Aktivitäten ging aus etwas mehr als der Hälfte der diesbezüglichen Angaben hervor (Tab. 4). Eine Veränderung der Qualität wurde seltener angegeben, wovon drei Viertel eine Verbesserung empfanden. Der Großteil der restlichen Angaben bezog sich auf eine Abnahme oder Verschlechterung bestimmter sexueller Aktivitäten (weniger sexuelle Aktivitäten außerhalb fester Partnerschaften, erschwertes Kennenlernen neuer Sexpartner_innen und Ausleben von Sexualität, gesteigerte sexuelle Frustration bei ausbleibender Befriedigung sexuellen Verlangens, weniger Sexpartner_innen). Wenige Personen berichteten von neuen Sexpartner_innen. Weiterhin berichteten einige Teilnehmer_innen über eine veränderte Motivation für sexuelle Handlungen („Man hat den Partner 24/7 gesehen, da war das sexuelle Zusammenkommen eher spannungslösend“; Z248) und Veränderungen in der Sexroutine („Durch Homeschooling und mehr Aufmerksamkeit und Unsicherheiten der Kinder wurden allerdings sexuelle Handlungen mehr in die Abend- und Nachtstunden verlagert“; Z135).

**Table 4:** 

	∑
**Veränderung in der Quantität sexueller Kontakte**	40
– 62,5 % Zunahme	
– 37,5 % Abnahme	
**Veränderung in der Qualität sexueller Kontakte**	8
– 75 % Zunahme	
– 25 % Abnahme	
**Reduktion sexueller Aktivitäten außerhalb fester Partnerschaften**	6
**Erschwertes Kennenlernen von Sexpartner_innen**	5
**Neue Sexpartnerschaft**	5
**Erschwertes Ausleben von Sexualität**	4
**Veränderte Motivation für Sex**	4
**Gesteigerte sexuelle Frustration**	4
**Weniger Sexpartner_innen**	2
**Veränderungen in der Sexroutine**	2

#### Sexuelle Interessen und Einstellungen

Als häufigste Unterkategorie von Veränderungen in sexuellen Interessen und/oder Einstellungen wurde eine veränderte Frequenz, meist Zunahme, sexueller Fantasien benannt (Tab. 5). Inhaltlich betrachtet kam es bei den Fantasien ebenfalls zu Veränderungen (z. B. softere Fantasien, Zunahme nicht ausgelebter sexueller Interessen). Ferner schilderten einige Teilnehmer_innen eine intensivere Beschäftigung mit den Themen Sexualität und/oder Partnerschaft. Das Thema Sexualität nahm in einem kleinen Teil der Stichprobe einen höheren Stellenwert ein, und es wurde von mehr sexueller Experimentierfreude berichtet. Ein Proband berichtete von Insuffizienzgefühlen in seiner männlichen Rolle: „Ich habe in der Krise einen vollständigen Rückzug sämtlicher Personen erlebt, an denen ich intimes Interesse gehabt hätte, und das Gefühl, als Mann vollständig nutz- und funktionslos zu sein“ (Z124).

**Table 5:** 

	∑
**Veränderung der Häufigkeit sexueller Fantasien**	15
– 80 % Zunahme	
– 20 % Abnahme	
**Intensivere Beschäftigung mit Sexualität/Partnerschaft**	12
**Mehr Experimentierfreude**	6
**Inhaltliche Veränderung sexueller Fantasien**	5
**Höherer Stellenwert von Sexualität**	5
**Gesteigerte sexuelle Offenheit**	3
**Zunahme nicht ausgelebter sexueller Interessen**	1
**Insuffizienzgefühle in männlicher Rolle**	1

#### Virtuelle sexuelle Aktivitäten

Virtuelle sexuelle Aktivitäten veränderten sich bei einem kleinen Anteil der Teilnehmer_innen besonders auf quantitativer Ebene (gesteigerter Pornografiekonsum, gesteigerte Nutzung von Sexting/Telefonsex und Video‑/Cybersex/Chaturbate). Auf qualitativer Ebene berichtete je eine Person über den Konsum softerer Pornografiegenres und über eine veränderte Motivation, Pornografie zu konsumieren („De[n] Konsum von Pornografie … habe ich häufig als Ablenkung von Langeweile empfunden“; Z9; Tab. 6).

**Table 6:** 

	∑
Gesteigerter Pornografiekonsum	5
Gesteigerte Nutzung von Sexting/Telefonsex	4
Gesteigerte Nutzung von Video‑/Cybersex/Chaturbate	2
Softere Pornografiegenres	1
Veränderte Motivation, Pornografie zu konsumieren	1

#### Dating

Als eine weitere Oberkategorie bildeten sich Veränderungen im Dating-Verhalten heraus (Tab. 7). Proband_innen berichteten von mehr Zurückhaltung im Dating allgemein sowie in Bezug auf sexuelle Kontakte zu fremden Personen, was beispielsweise auf eine „innere Blockade“ (Z137) oder ein schlechtes Gewissen aufgrund der Kontaktbeschränkungen zurückgeführt wurde. Weiterhin wurde von durch die Kontaktbeschränkungen bedingten Hürden berichtet, potenzielle Partner_innen auf sozialen Events kennenzulernen. Als eine weitere Veränderung wurde eine offenere Haltung gegenüber Online-Dating genannt: „Ich bin offener geworden (vielleicht auch weniger kritisch oder misstrauisch) in Bezug auf Online-Dating“ (Z25).

**Table 7:** 

	∑
Zurückhaltung im Dating	3
Offenere Haltung gegenüber Online-Dating	3
Hürden durch Kontaktbeschränkungen, Personen zu daten	2
Zurückhaltung bezüglich sexueller Kontakte zu fremden Personen	2

#### Sexuelle Probleme und Funktionsstörungen

Zwei Personen informierten über eine Belastung durch die Zunahme ihres sexuellen Verlangens und eine durch dessen Abnahme. Erregungs- und Orgasmusprobleme wurden von je einer Person berichtet („[Ich habe] seit einigen Wochen leichte Erektionsprobleme, die vorher nicht existierten“; Z149; Tab. 8).

**Table 8:** 

	∑
**Belastung durch Veränderungen sexuellen Verlangens**	3
– 66,7 % Zunahme	
– 33,3 % Abnahme	
**Erregungsprobleme**	1
**Orgasmusprobleme**	1

## Diskussion

Die Ergebnisse der vorliegenden Studie zeigen, dass etwa drei Viertel der befragten Personen während der pandemiebedingten Kontaktbeschränkungen Veränderungen in ihren sexuellen Interessen und Erfahrungen erlebt haben. Inhaltlich wurden Veränderungen in verschiedenen Bereichen beschrieben: sexuelles Verlangen und Erregbarkeit, Partnerschaft, sexuelle Aktivitäten, sexuelle Interessen und Einstellungen, Dating und sexuelle Probleme bzw. Funktionsstörungen.

Aus einer weiteren Ausdifferenzierung und Quantifizierung dieser Kategorien konnte geschlossen werden, dass bei denjenigen, die über eine jeweilige Veränderung berichteten, häufiger ein verstärktes sexuelles Verlangen als ein reduziertes sexuelles Verlangen berichtet wurde. Diese Tendenz zeigte sich (wenngleich quantitative und qualitative Studien nur begrenzt miteinander vergleichbar sind) auch in der eingangs beschriebenen EU- und türkeiweiten Studie (Stuhlhofer et al. in Revision). Zunahmen fanden sich ebenfalls bezüglich des Verlangens nach Nähe, Intimität und Körperlichkeit sowie in der Masturbationsfrequenz, wobei vorherige quantitative Studien ein eher heterogenes Bild zeichneten (Ibarra et al. 2020; Günther-Bel et al. 2020; Luetke et al. 2020).

Im Bereich der Partnerschaft wurde von einer Zunahme der mit dem/der Partner_in verbrachten Zeit und einer zunehmenden emotionalen Intimität in Partnerschaften berichtet. Dies wurde auch in einer vorherigen quantitativen Studie (Günther-Bel et al. 2020) berichtet, scheint aber von verschiedenen Faktoren, wie dem Zusammenleben mit Kindern, abzuhängen. Darüber hinaus wurde eine Veränderung der gelebten Partnerschaftskonzepte hinsichtlich der Offenheit nichtmonogamer Beziehungskonzepte durch die Kontaktbeschränkungen berichtet. In Bezug auf sexuelle Kontakte berichteten mehr Personen, die Veränderungen angaben, von häufigerem und besserem Sex als von weniger und schlechterem Sex. Deutlich wurde außerdem ein erschwertes Zustandekommen von sexuellen Kontakten außerhalb bereits bestehender partnerschaftlicher oder sexueller Beziehungen. Ein größerer Anteil an Personen, die eine veränderte Frequenz sexueller Fantasien nannten, berichtete von einer Zunahme, ein geringerer Teil von einer Abnahme. Zudem zeigte sich, dass während der Kontaktbeschränkungen eine intensivere Beschäftigung mit den Themen Sexualität und Partnerschaft erfolgte. Im Bereich virtueller sexueller Aktivitäten wurde ein gesteigerter Konsum von Pornografie und digitaler Kommunikation berichtet. Wenngleich dies nur von einer kleinen Gruppe mitgeteilt wurde, finden sich diese Tendenzen auch in den Angaben großer Internetpornografieanbieter (Pornhub 2020). Auch in der Kategorie Dating wurde während der Kontaktbeschränkungen eine verstärkte Offenheit gegenüber virtuellen Möglichkeiten beschrieben, und gleichzeitig mehr Zurückhaltung gegenüber persönlichen Dating-Kontakten. In Bezug auf sexuelle Probleme zeigten sich häufiger eine Zunahme der Belastung durch Veränderungen des sexuellen Verlangens und seltener eine Abnahme des Leidensdrucks.

Limitierend soll hervorgehoben werden, dass die vorliegende Stichprobe nicht repräsentativ ist. Die vorliegende Stichprobe erwies sich beispielsweise als überdurchschnittlich gebildet, großstädtisch und aus Singles bestehend. Es ist anzunehmen, dass eher sexuell aufgeschlossene Menschen oder diejenigen mit einem in diesem Bereich vorhandenen Leidensdruck sich zur Teilnahme an der Studie bereit erklärt haben. Zudem sind Antworttendenzen nach sozialer Erwünschtheit möglich. Des Weiteren kann die verwendete qualitative Analyse zwar dem Anspruch gerecht werden, Hypothesen über relevante Kategorien zu generieren, nicht jedoch über deren klinische Relevanz, wofür eine quantitative Folgestudie angemessen wäre. In künftigen Studien wäre es sinnvoll, die gewonnenen Kategorien in einer möglichst repräsentativeren Stichprobe zu quantifizieren und Moderatoren für die beschriebenen Veränderungen herauszuarbeiten, um zu klären, ob diese beispielsweise durch bestimmte Geschlechter oder Beziehungskonzepte prädiziert werden.

Die vorliegenden Ergebnisse ergänzen die bisher insgesamt noch sehr inhomogene Datenlage zu pandemiebedingten Veränderungen von sexuellen Interessen und Erfahrungen und bieten weitere Anhaltspunkte, welche Konfliktbereiche in Therapie- und Beratungssettings angesprochen werden können. Da sexuelle Probleme in der Praxis ohnehin zu selten angesprochen werden, Patient_innen sich aber die aktive Ansprache wünschen, sollten beratende und psychotherapeutisch Tätige wissen, welche Änderungen durch die Pandemie zu erwarten sein könnten. Hier hat sich durch die offene Frage in dieser Studie eine Reihe neuer Erkenntnisse gezeigt, die über die bisherigen quantitativen Studien hinausgehen. Mit dem Ziel, die sexuelle und psychische Gesundheit zu fördern, empfiehlt es sich, Sexualität in der alltäglichen Praxis routinemäßig anzusprechen (Dekker et al. 2020) und beispielsweise ökonomische Screeninginstrumente für sexuelle Dysfunktionen einzusetzen (Briken et al. 2020).

## Fazit für die Praxis

- Ein Großteil der Menschen erlebt durch die pandemiebedingten Kontaktbeschränkungen veränderte sexuelle Interessen oder Erfahrungen.
- Diese Veränderungen können mit persönlichen oder partnerschaftlichen Konflikten einhergehen und gleichzeitig wichtige Ressourcen im Umgang mit Kontaktbeschränkungen darstellen.
- Um sexuelle Gesundheit künftig routiniert mitzudenken, sollten insbesondere die von Veränderungen betroffenen Bereiche der Sexualität und Partnerschaft in Therapie- und Beratungssettings aktiv angesprochen werden.
